# Characteristics and outcomes in elderly patients with non-valvular atrial fibrillation and high bleeding risk: subanalysis of the J-RHYTHM Registry

**DOI:** 10.1007/s00380-023-02343-9

**Published:** 2023-12-16

**Authors:** Eitaro Kodani, Hiroshi Inoue, Hirotsugu Atarashi, Ken Okumura, Takeshi Yamashita, Hideki Origasa, the J-RHYTHM Registry Investigators

**Affiliations:** 1 Department of Cardiovascular Medicine and Cardiology, Nippon Medical School Tama Nagayama Hospital, 1-7-1 Nagayama, Tama-shi, Tokyo, 206-8512 Japan; 2 Saiseikai Toyama Hospital, Toyama, Japan; 3 AOI Hachioji Hospital, Tokyo, Japan; 4 Saiseikai Kumamoto Hospital, Kumamoto, Japan; 5 The Cardiovascular Institute, Tokyo, Japan; 6 The Institute of Statistical Mathematics, Tokyo, Japan

**Keywords:** Atrial fibrillation, Elderly, High bleeding risk, Warfarin, All-cause death

## Abstract

Recently, a once-daily dose of edoxaban (15-mg) has been approved for stroke prevention in non-valvular atrial fibrillation (NVAF) patients aged ≥ 80 years, in whom standard oral anticoagulants are not recommended because of high bleeding risk (HBR), based on the ELDERCARE-AF trial. However, information regarding the characteristics and clinical outcomes among such patients is limited. Thus, this study aimed to clarify the characteristics and event rates in elderly patients with NVAF and HBR defined by the ELDERCARE-AF criteria. Of the 7406 NVAF outpatients included in the J-RHYTHM Registry, 60 patients with creatinine clearance (CrCl) < 15 mL/min were excluded. The remaining 7346 patients (age, 69.7 ± 9.9 years; men, 70.9%; warfarin use, 78.7%) were divided into three groups: Group 1, aged < 80 years (*n* = 6165); Group 2, aged ≥ 80 years without HBR (*n* = 584); and Group 3, aged ≥ 80 years with HBR (at least one of the followings; CrCl, 15–30 mL/min, history of bleeding, body weight ≤ 45 kg, and antiplatelet use) (*n* = 597, eligible for 15-mg edoxaban). Patients in Group 3 had a higher prevalence of comorbidities, and therefore, both higher thromboembolic and bleeding risk scores than in the other groups. During the 2-year follow-up period, the incidence rates (per 100 person-years) of thromboembolism in Groups 1, 2, and 3 were 0.7, 1.5, and 2.1 (*P* < 0.001), major hemorrhage, 0.8, 1.2, and 2.0 (*P* < 0.001), and all-cause death, 0.8, 2.6, and 4.6 (*P* < 0.001), respectively. Adjusted hazard ratios of Group 3 were 1.64 (95% confidence interval 0.89–3.04, *P* = 0.116) for thromboembolism, 1.53 (0.85–2.72, *P* = 0.154) for major hemorrhage, and 1.84 (1.19–2.85, *P* = 0.006) for all-cause death compared with Group 1. The NVAF Patients aged ≥ 80 years with HBR defined by the ELDERCARE-AF criteria were certainly at a higher adverse event risk, especially for all-cause death. *Clinical trial registration*: The J-RHYTHM Registry is registered in the University Hospital Medicine Information Network (UMIN) Clinical Trials Registry (unique identifier: UMIN000001569) http://www.umin.ac.jp/ctr/.

**Supplementary Information:**

The online version contains supplementary material available at 10.1007/s00380-023-02343-9.

## Introduction

Atrial fibrillation (AF) is the most common sustained arrhythmia in adult, particularly in those of advanced age. The prevalence of AF in the population aged ≥ 80 years is reportedly 7 to 14% in Western countries [1] and 2 to 3% in Japan [2]. The number of subjects with AF is expected to increase further with the aging of society [1, 2]. AF is a major risk factor for ischemic stroke and systemic embolism (SE) [3, 4] and aging itself is a potent risk factor for stroke [5]. Thus, anticoagulation therapy is generally recommended to prevent AF-related thromboembolism in patients with AF, especially in elderly patients. Because anticoagulation therapy has a trade-off between prophylaxis of thromboembolism and an increase in the bleeding risk, physicians often hesitate to prescribe an oral anticoagulant to avoid bleeding complications, especially in very elderly patients with a high bleeding risk. Although non-vitamin K antagonist oral anticoagulants (NOACs) reportedly have a lower bleeding risk than well-controlled warfarin [6], several patients who are ineligible candidates for approved doses of NOACs still exist in actual clinical settings. Recently, a once-daily dose of edoxaban (15-mg) has been approved in Japan for stroke prevention in non-valvular AF (NVAF) patients aged ≥ 80 years, in whom standard anticoagulation therapy is not recommended because of high bleeding risk, based on the Edoxaban Low-Dose for Elder Care Atrial Fibrillation Patients (ELDERCARE-AF) trial [7]. However, information regarding the characteristics and clinical outcomes among such patients remains limited in Japan [8, 9]. Therefore, we elucidated the characteristics and event rates in NVAF patients aged ≥ 80 years with high bleeding risk, who are now eligible for once-daily 15-mg edoxaban, and the impact of high bleeding risk for adverse events, using data of the J-RHYTHM Registry [10–12]. In addition, since the effectiveness and safety of edoxaban 15-mg versus placebo for very elderly NVAF patients with high bleeding risk were investigated in the ELDERCARE-AF trial [7], we elucidated event rates in patients who treated with warfarin and without oral anticoagulant in this study population.

## Methods

### Study design of the J-RHYTHM Registry

The J-RHYTHM Registry was conducted as a nationwide prospective observational study to investigate the status of warfarin therapy and optimal warfarin therapy in Japanese patients with AF [10]. The study design and baseline patient characteristics have been reported elsewhere [10, 11]. The study protocol conformed to the Declaration of Helsinki and was approved by the ethics committee of Japanese Society of Electrocardiology (H20-01) and each participating institution. Written informed consent was obtained from all participants at the time of enrollment. A consecutive series of outpatients with AF of any type were enrolled from 158 institutions, regardless of anticoagulant use. All the drugs and their dosages were selected at the discretion of the treating physicians. Patients with valvular AF (mechanical prosthetic valve and/or mitral stenosis) were excluded.

The anticoagulation intensity in patients treated with warfarin was determined at the time of enrollment (baseline) and at each visit during the follow-up period using the prothrombin time-international normalized ratio (PT-INR). The overall quality of anticoagulation therapy with warfarin during the follow-up period was evaluated using the time in therapeutic range calculated by the Rosendaal method [13]. The target PT-INR level was set at 1.6–2.6 for elderly patients aged ≥ 70 years and at 2.0–3.0 for patients aged < 70 years according to the Japanese guidelines [14].

### Definition of high bleeding risk and grouping of patients

Patients with severe renal insufficiency with creatinine clearance (CrCl) < 15 mL/min were excluded from this subanalysis, as in the ELDERCARE-AF trial. High bleeding risk was defined as at least one of the followings: (i) low CrCl of 15–30 mL/min, (ii) history of bleeding in a critical area or organ or gastrointestinal bleeding, (iii) low body weight (≤ 45 kg), (iv) continuous use of nonsteroidal anti-inflammatory drugs (NSAIDs), and (v) current use of an antiplatelet drug, according to the ELDERCARE-AF trial (Table 1) [7].

**Table 1:** Eligible patients for once-daily 15-mg edoxaban

Factors	Frequency in group 3 (*n* = 597)
1. Non-valvular AF patients aged ≥ 80 years	
2. Ineligible candidates for standard anticoagulation therapy at the recommended therapeutic strength of warfarin (PT-INR 1.6–2.6) or at approved doses of NOACs because of one or more high bleeding risks as follows	
(i) Low creatinine clearance (15–30 mL/min)	188 (31.5%)
(ii) History of bleeding in a critical area or organ or gastrointestinal bleeding	59 (9.9%)
(iii) Low body weight (≤ 45 kg)	201 (33.7%)
(iv) Continuous use of nonsteroidal anti-inflammatory drugs	Not evaluated
(v) Current use of an antiplatelet drug	367 (61.5%)

*AF* atrial fibrillation; *PT-INR* prothrombin time-international normalized ratio; *NOACs*, non-vitamin K antagonist oral anticoagulants

The patients were divided into three groups based on age and the presence of high bleeding risk: patients aged < 80 years (Group 1), those aged ≥ 80 years without high bleeding risk (Group 2), and those aged ≥ 80 years with one or more high bleeding risks (Group 3, patients eligible for 15-mg edoxaban) (Table 1). Because a CHADS_2_ score ≥ 2 is not an essential criterion for the use of once-daily 15-mg edoxaban in the current package insert of edoxaban, patients with CHADS_2_ score of 1 and high bleeding risk were assigned to Group 3.

### Follow-up and endpoints

Patients were followed up for 2 years or until an event occurred, whichever occurred first. The primary endpoints were thromboembolism, including symptomatic ischemic stroke, transient ischemic attack (TIA), and SE; major hemorrhage, including intracranial hemorrhage (ICH), gastrointestinal bleeding, and other hemorrhages requiring hospitalization; and all-cause and cardiovascular deaths. The diagnostic criteria for each event have been described elsewhere [10, 11]. The 2-year event rates in each group were calculated and compared between the subgroups of patients who were treated with warfarin and those who were not.

### Statistical analyses

Data are presented as mean ± standard deviation (SD) or number (percentage). To compare the patient characteristics and event rates among three groups, the analysis of variance for continuous variables or Chi-square test for categorical variables was performed. The differences in event rates between the two subgroups were analyzed using the Chi-square test or Fisher’s exact test, as appropriate. Cumulative event-free rates were expressed using Kaplan–Meier curves and compared among the three groups using a log-rank test. Univariable and multivariable analyses using Cox proportional hazards models were performed to investigate the influence of age and high bleeding risk on adverse events. Hazard ratios (HRs) and 95% confidence intervals (CIs) were calculated using Group 1 as a reference. The Cox proportional hazards assumption was verified using a log–log survival curve for all study outcomes. Explanatory variables for multivariable analysis were adopted based on the significantly different variables in patient characteristics and medications among three groups (Table 2), including age, sex, congestive heart failure, hypertension, diabetes mellitus, history of stroke or TIA, coronary artery disease, cardiomyopathy, congenital heart disease, chronic obstructive pulmonary disease, hyperthyroidism, warfarin use, AF type, and hemoglobin levels, except for components of the high bleeding risk. Two-tailed *P*-values of < 0.05 were considered statistically significant. All statistical analyses were performed using the SPSS software (version 23.0; IBM Corporation, Armonk, NY, USA).

**Table 2:** Patient characteristics and medications

	Group 1 (< 80 years)	Group 2 (≥ 80 years without HBR)	Group 3 (≥ 80 years with HBR)	*P*-value
Number of patients	6165	584	597	
Age, years	67.1 ± 8.6	82.6 ± 2.6	83.7 ± 3.4	< 0.001
Sex, men	4488 (72.8%)	391 (67.0%)	327 (54.8%)	< 0.001
Body weight, kg	63.9 ± 12.6	58.7 ± 8.7	51.4 ± 11.0	< 0.001
Body mass index, kg/m^2^	23.9 ± 4.0	23.2 ± 3.2	21.3 ± 3.6	< 0.001
Type of atrial fibrillation				
Paroxysmal	2433 (39.5%)	196 (33.6%)	184 (30.8%)	< 0.001
Persistent	932 (15.1%)	77 (13.2%)	63 (10.6%)	
Permanent	2800 (15.4%)	311 (53.3%)	350 (58.6%)	
Comorbidities				
Coronary artery disease	608 (9.9%)	32 (5.5%)	127 (21.3%)	< 0.001
Cardiomyopathy	557 (9.0%)	35 (6.0%)	34 (5.7%)	0.002
HCM	227 (3.7%)	19 (3.3%)	15 (2.5%)	0.311
DCM	330 (5.4%)	16 (2.7%)	19 (3.2%)	0.002
Congenital heart disease	90 (1.5%)	4 (0.7%)	2 (0.3%)	0.027
COPD	94 (1.5%)	18 (3.1%)	19 (3.2%)	0.001
Hyperthyroidism	124 (2.0%)	3 (0.5%)	3 (0.5%)	0.002
Risk factors for stroke				
Heart failure	1527 (24.8%)	214 (36.6%)	276 (46.2%)	< 0.001
Hypertension	3671 (59.5%)	376 (64.4%)	388 (65.0%)	< 0.001
Age (≥ 75 years)	1351 (21.9%)	584 (100%)	597 (100%)	0.446
Diabetes mellitus	1125 (18.2%)	100 (17.1%)	119 (19.9%)	< 0.001
Stroke/TIA	778 (12.6%)	102 (17.5%)	132 (22.1%)	< 0.001
CHADS_2_ score	1.5 ± 1.1	2.5 ± 1.1	2.8 ± 1.2	< 0.001
CHA_2_DS_2_-VASc score	2.5 ± 1.5	3.9 ± 1.3	4.4 ± 1.3	< 0.001
HAS-BLED score	1.4 ± 1.0	1.6 ± 0.7	2.3 ± 0.9	< 0.001
Systolic BP, mmHg	125.9 ± 15.9	126.5 ± 16.4	125.8 ± 17.3	0.728
Diastolic BP, mmHg	74.3 ± 17.7	70.4 ± 11.1	69.3 ± 11.8	< 0.001
Heart rate, /min	72.4 ± 13.2	73.3 ± 12.5	73.5 ± 13.4	0.121
Creatinine clearance, mL/min	73.9 ± 26.2	49.7 ± 13.4	37.1 ± 13.8	< 0.001
Hemoglobin, g/dL	13.9 ± 1.7	13.0 ± 1.7	12.3 ± 1.7	< 0.001
Medications				
Warfarin	5333 (86.5%)	549 (94.0%)	470 (78.7%)	< 0.001
Dosage, mg/day	3.0 ± 1.2	2.4 ± 0.9	2.2 ± 1.0	< 0.001
PT-INR	1.91 ± 0.49	1.90 ± 0.48	1.87 ± 0.52	0.202
TTR*, % (*n* = 6016)	57.4 ± 29.4	72.7 ± 25.0	66.3 ± 25.5	< 0.001
Antiplatelet	1546 (25.1%)	0 (0.0%)	367 (61.5%)	< 0.001
Aspirin	1377 (22.3%)	0 (0.0%)	282 (47.2%)	< 0.001
Others	310 (5.0%)	0 (0.0%)	110 (18.4%)	< 0.001
Warfarin + antiplatelet	1081 (17.5%)	0 (0.0%)	260 (43.6%)	< 0.001
ARB/ACE-I	3253(52.8%)	345 (53.9%)	323 (54.9%)	0.537
Statins	1539 (25.0%)	103 (17.6%)	142 (23.8%)	< 0.001

Data are number of patients (%) or mean ± standard deviation

*HBR* high bleeding risk; *HCM* hypertrophic cardiomyopathy; *DCM* dilated cardiomyopathy; *COPD* chronic obstructive pulmonary disease; *TIA* transient ischemic attack; *CHADS*_*2*_ congestive heart failure, hypertension, age ≥ 75 years, diabetes mellitus, and history of stroke or TIA; *CHA*_*2*_*DS*_*2*_*-VASc* additionally, vascular disease (coronary artery disease), age 65–74 years, and female sex; *HAS-BLED* hypertension (systolic BP ≥ 140 mmHg), abnormal renal/liver function, stroke, bleeding history or predisposition, labile INR (episodes of INR ≥ 3.5), elderly (age > 65 years), drugs (use of antiplatelets)/alcohol concomitantly; *PT-INR* prothrombin time-international normalized ratio; *TTR* time in therapeutic range; *ARB* angiotensin II receptor blacker; ACE-I, angiotensin converting enzyme inhibitor

*Target PT-INR was 2.0–3.0 (< 70 years) or 1.6–2.6 (≥ 70 years)

## Results

Of a total of 7937 patients with AF enrolled in the J-RHYTHM Registry, 421 (5.3%) with valvular AF were excluded and 110 (1.5%) were lost to follow-up. Of the remaining 7406 patients with NVAF, 60 (0.8%) patients with CrCl < 15 mL/min were excluded. Consequently, 7346 patients (age, 69.7 ± 9.9 years; men, 70.9%) were included in this subanalysis and divided into three groups. The number of patients in each group was 6165, 584, and 597, respectively. The frequency of each high bleeding risk in Group 3 is shown in Table 1.

### Patient characteristics and medications

The clinical characteristics of each group are shown in Table 2. The prevalence of permanent AF, coronary heart disease, chronic obstructive pulmonary disease, heart failure, hypertension, diabetes mellitus, prior stroke or TIA, and antiplatelet use was significantly different among the three groups. Both thromboembolic risk (CHADS_2_ and CHA_2_DS_2_-VASc scores) and bleeding risk (HAS-BLED score) therefore significantly differed among the three groups (Table 2). In addition, body weight, body mass index, diastolic blood pressure, CrCl, hemoglobin levels, warfarin dosage, and PT-INR were also significantly different among the groups.

### Event rates

**Fig. 1:**
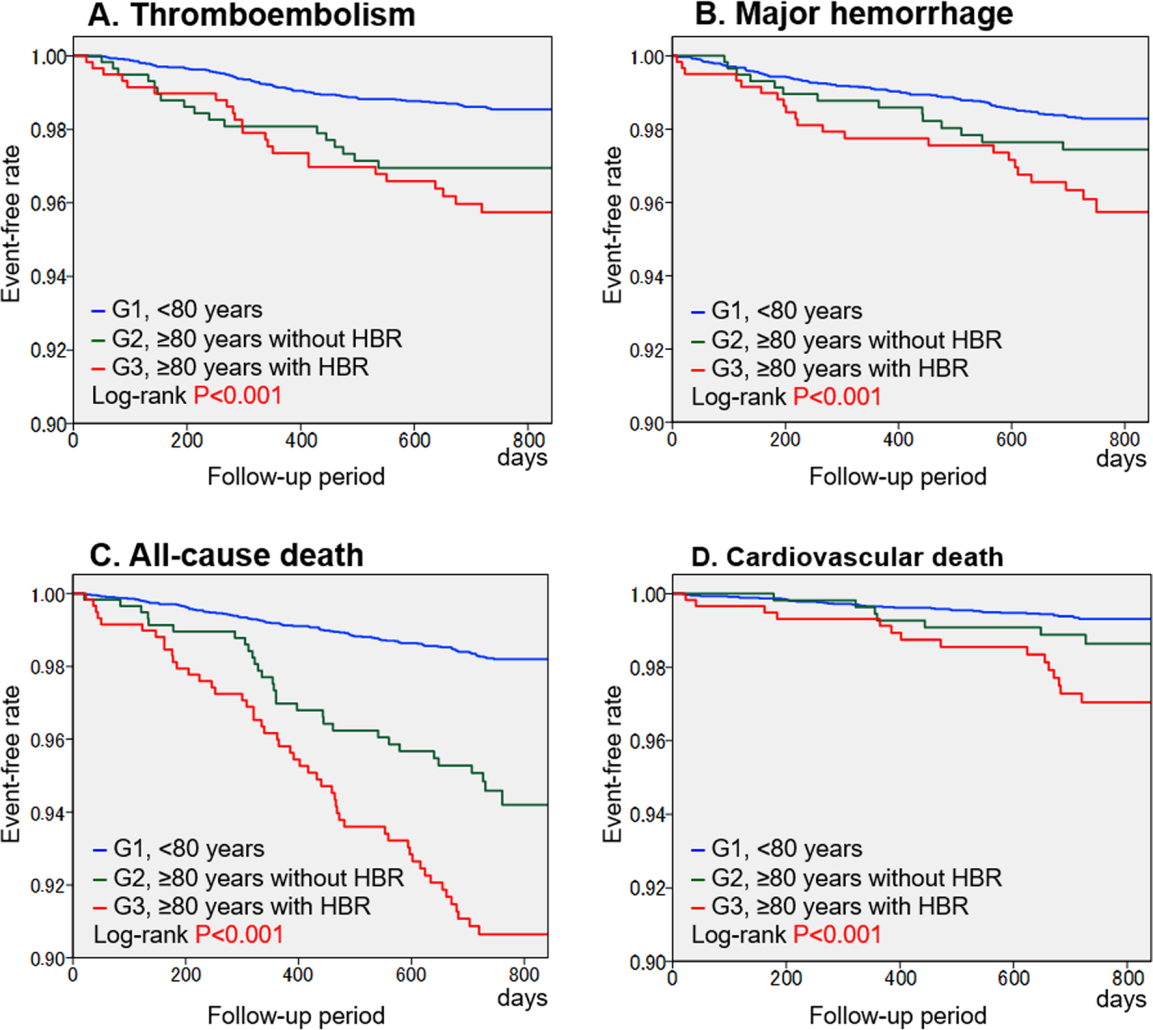
Kaplan–Meier curves for thromboembolism (**A**), major hemorrhage (**B**), all-cause death (**C**), and cardiovascular death (**D**). *P*-values: comparison among groups using the log-rank test. *G* Group; *HBR* high bleeding risk

During the 2-year follow-up period (median: 753 days), thromboembolism, major hemorrhage, all-cause death, and cardiovascular death occurred in 125 (1.7%), 137 (1.9%), 185 (2.5%), and 62 (0.8%) patients, respectively. The corresponding incidence rates of these events were 0.85, 0.94, 1.26, and 0.42 /100 person-years, respectively, during a follow-up period of 14,641 person-years. The crude numbers and rates of adverse events in each group are summarized in Table 3. The rates of thromboembolism; major hemorrhage, including ICH and gastrointestinal bleeding; all-cause death; and cardiovascular death were significantly different among the three groups and highest in Group 3 (Table 3). In the Kaplan–Meier curves, the cumulative event-free rates for all adverse events were significantly different among the three groups (*P* < 0.001 for all, log-rank test), with the lowest event-free rates in Group 3 (Fig. 1).

**Table 3:** Crude event numbers and rates during two-year follow-up period

	Group 1 (< 80 years)	Group 2 (≥ 80 years without HBR)	Group 3 (≥ 80 years with HBR)	*P*-value
Number of patients	6165	584	597	
Thromboembolism	85 (1.4%)	17 (2.9%)	23 (3.9%)	< 0.001
Major hemorrhage	101 (1.6%)	14 (2.4%)	22 (3.7%)	< 0.001
Intracranial hemorrhage	36 (0.6%)	8 (1.4%)	9 (1.5%)	0.006
Gastrointestinal bleeding	31 (0.5%)	4 (0.7%)	9 (1.5%)	0.010
Other bleeding or unknown	34 (0.6%)	2 (0.3%)	4 (0.7%)	0.734
All-cause death	104 (1.7%)	30 (5.1%)	51 (8.5%)	< 0.001
Cardiovascular death	40 (0.6%)	7 (1.2%)	15 (2.5%)	< 0.001

Data are number of patients (%)

*HBR* High bleeding risk

### Event risk in patients with high bleeding risk

The unadjusted HRs for thromboembolism and all-cause death in Group 2 and for all adverse events in Group 3 were significantly higher than those in Group 1 on univariable analysis (Table 4). After adjusting for confounding factors in the multivariable analysis, the adjusted HR was significantly higher only for all-cause death in Group 3 than in Group 1 (Table 4).

**Table 4:** Influence of age (≥ 80 years) and high bleeding risk on adverse events (Cox proportional hazards analysis)

	Thromboembolism		Major hemorrhage		All-cause death		Cardiovascular death	
	HR (95% CI)	*P*-value	HR (95% CI)	*P*-value	HR (95% CI)	*P*-value	HR (95% CI)	*P*-value
Univariable								
Group 1 (< 80 years)	1.00 (reference)		1.00 (reference)		1.00 (reference)		1.00 (reference)	
Group 2 (≥ 80 years without HBR)	2.17 (1.29–3.66)	0.003	1.51 (0.86–2.64)	0.149	3.16 (2.10–4.74)	< 0.001	1.92 (0.86–4.28)	0.112
Group 3 (≥ 80 years with HBR)	2.97 (1.87–4.71)	< 0.001	2.39 (1.51–3.80)	< 0.001	5.45 (3.90–7.62)	< 0.001	4.18 (2.31–7.57)	< 0.001
Multivariable*								
Group 1 (< 80 years)	1.00 (reference)		1.00 (reference)		1.00 (reference)		1.00 (reference)	
Group 2 (≥ 80 years without HBR)	1.54 (0.79–3.00)	0.203	1.00 (0.52–1.94)	0.997	1.51 (0.91–2.52)	0.112	1.32 (0.52–3.36)	0.559
Group 3 (≥ 80 years with HBR)	1.64 (0.89–3.04)	0.116	1.53 (0.85–2.72)	0.154	1.84 (1.19–2.85)	0.006	1.49 (0.68–3.25)	0.317

*HR* Hazard ratio, *CI* confidence interval, *HBR* high bleeding risk

*Adjusted for age, sex, congestive heart failure, hypertension, diabetes mellitus, history of stroke or transient ischemic attack, coronary artery disease, cardiomyopathy, congenital heart disease, chronic obstructive pulmonary disease, hyperthyroidism, warfarin use, atrial fibrillation type, and hemoglobin level

### Incidence rates and warfarin use

**Fig. 2:**
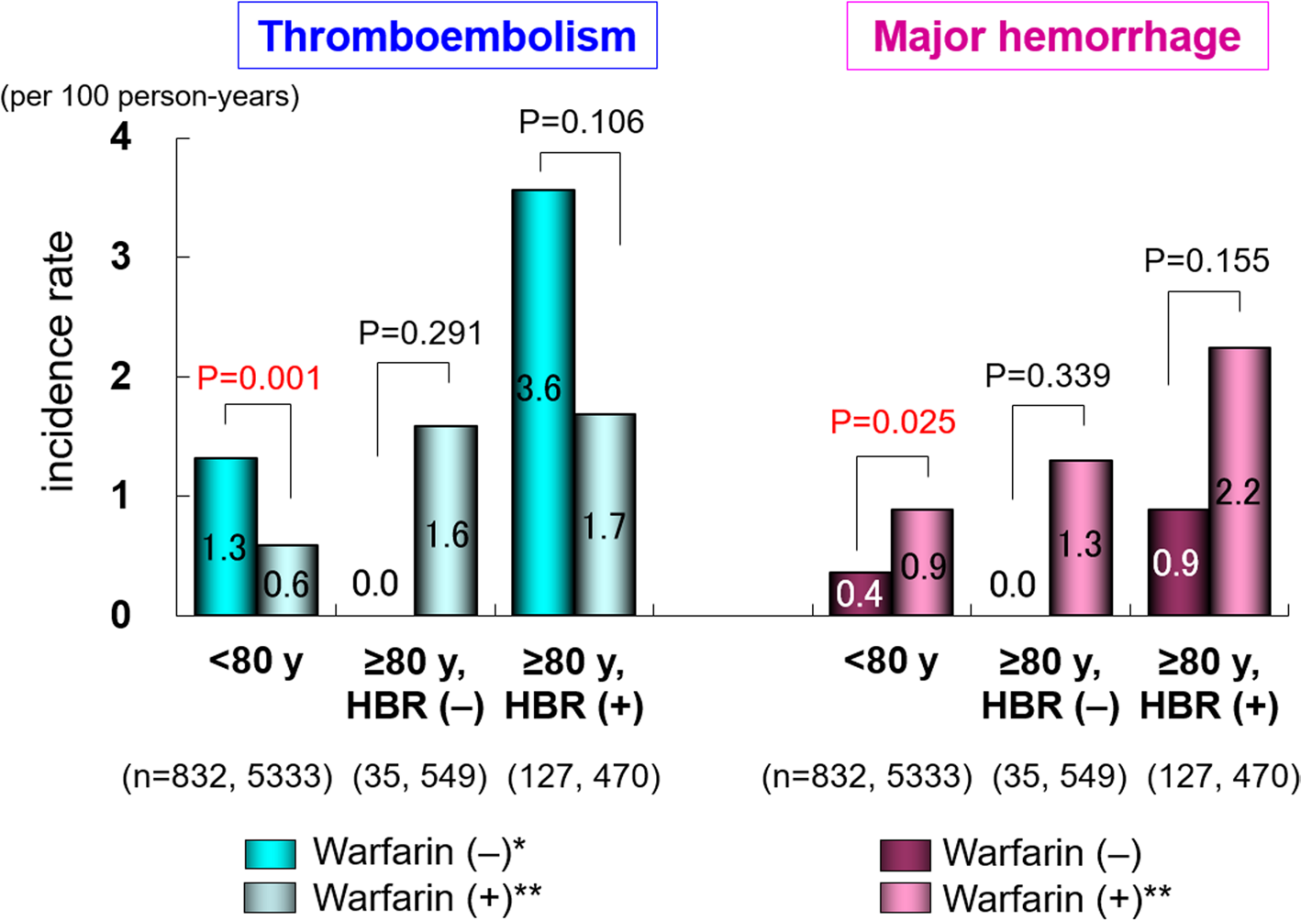
Incidence rates of thromboembolism and major hemorrhage in each group. *P*-values: comparison between patients with and without warfarin in each group. *P < 0.05, **P < 0.001. *HBR* High bleeding risk

**Fig. 3:**
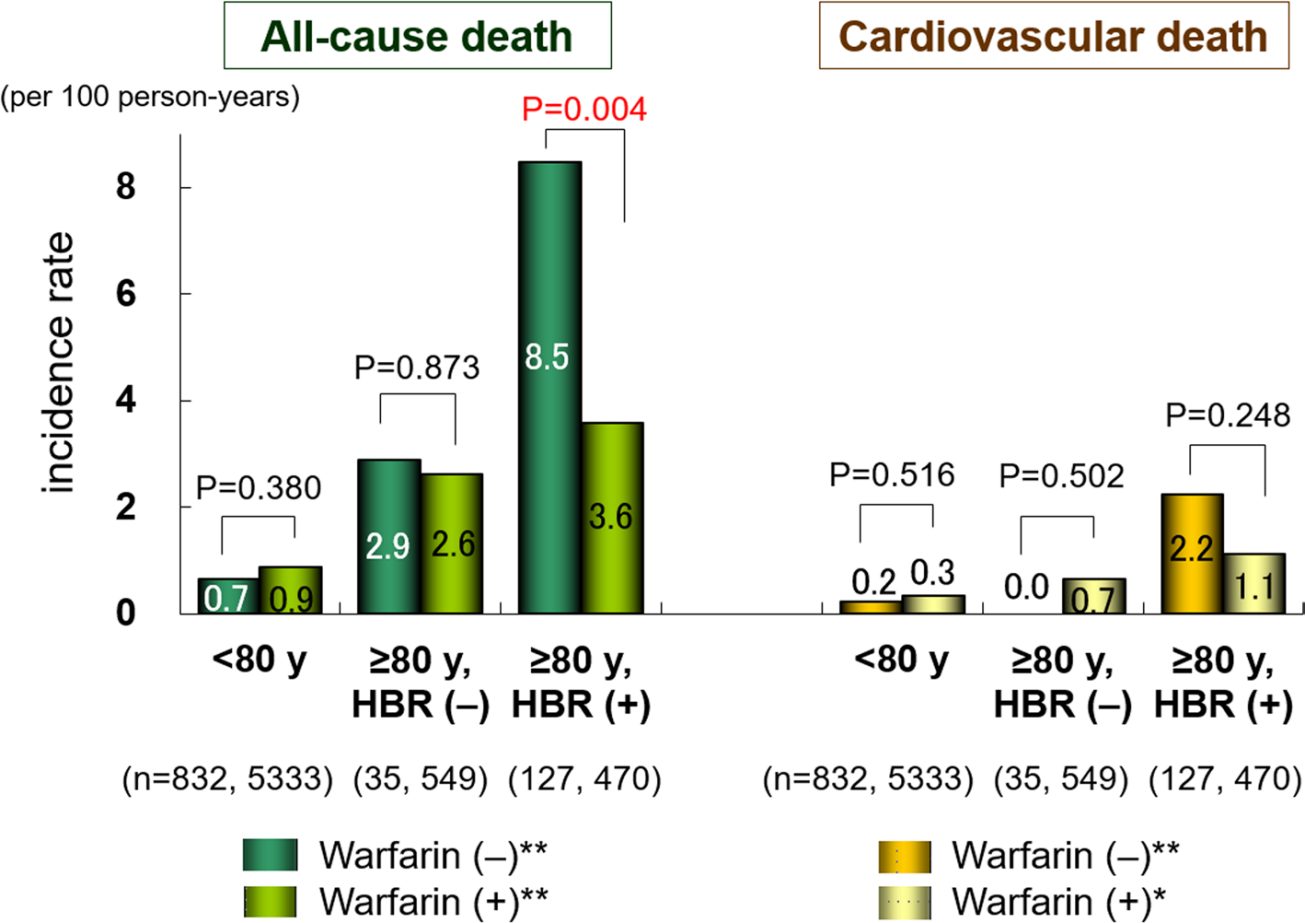
Incidence rates of all-cause and cardiovascular deaths in each group. *P*-values: comparison between patients with and without warfarin in each group. *P < 0.05, **P < 0.001. *HBR* High bleeding risk

Incidence rates of adverse events in the subgroups regarding warfarin use are shown in Figs. 2 and 3. In Group 3, the incidence rates (per 100 person-years) of thromboembolism in patients with versus without warfarin were 1.7 and 3.6 (*P* = 0.106); major hemorrhage, 2.2 and 0.9 (*P* = 0.155) (Fig. 2 and Supplementary Figure); all-cause death, 3.6 and 8.5 (*P* = 0.004), and cardiovascular death, 1.1 and 2.2 (*P* = 0.248) (Fig. 3), respectively.

## Discussion

In the present study, the high-bleeding-risk was defined following the criteria of ELDERCARE-AF trial [9], but not following the conventional bleeding risk scores, e.g., HAS-BLED score [15]. The major findings of this study are as follows. First, patients aged ≥ 80 years with a high bleeding risk (Group 3) had a higher prevalence of comorbidities, and therefore, higher CHADS_2_ and CHA_2_DS_2_-VASc scores, as well as higher HAS-BLED score. Indeed, the rates of all adverse events, not limited to major hemorrhage, were certainly higher in Group 3 than in the other groups. Second, the unadjusted HRs for all adverse events were significantly higher in Group 3; however, the adjusted HR was significantly higher only for all-cause death in Group 3 than in patients aged < 80 years. Third, in Group 3, warfarin use was associated with lower all-cause mortality.

### Characteristics of NVAF patients with high bleeding risk

In Group 3 of the present study, the current use of an antiplatelet drug was more prevalent (61.5%), followed by low body weight (≤ 45 kg, 33.7%), low CrCl (15–30 mL/min, 31.5%), and a history of bleeding (9.9%) among the five components of high bleeding risk (Table 1). Information regarding the continuous use of NSAIDs was not collected in this study. According to our previous report on the risk factors for bleeding in the J-RHYTHM Registry, prior bleeding, elderly (age > 65 years), labile INR, and antiplatelet use were independent risk factors for major bleeding among components of the HAS-BLED score [16]. In addition, low body weight, low CrCl values, and anemia were independent risk factors for all-cause death rather than for major hemorrhage [17–19]. In the present study, patients in Group 3 had lower body weight and CrCl levels according to the study design, and lower hemoglobin levels in addition to a higher prevalence of components of the conventional risk scores, as shown in Table 2. Inevitably, both thromboembolic risk (CHADS_2_ and CHA_2_DS_2_-VASc scores [20, 21]) and bleeding risk (HAS-BLED score [15]) in Group 3 were the highest among the three groups. These findings indicate that elderly patients with NVAF and a high bleeding risk defined by the ELDERCARE-AF criteria [7] also had a higher risk of thromboembolic events and all-cause death. Similar findings were reported in patients with acute myocardial infarction after percutaneous coronary intervention, in which the high bleeding risk criteria could be useful to identify patients at high ischemic risk along with high bleeding risk [22, 23].

The patient characteristics of Group 3 in the present study were similar to those of the ELDERCARE-AF trial [7] and the high-bleeding-risk group in a subanalysis of the All Nippon Atrial Fibrillation in the Elderly (ANAFIE) Registry [9]. Among the clinical variables, the frequency of comorbidities and CHADS_2_ and CHA_2_DS_2_-VASc scores were slightly lower in the present study than in the ELDERCARE-AF trial [7] and subanalysis of the ANAFIE Registry [9] (Supplementary Table 1). In contrast, the frequencies of non-paroxysmal AF and antiplatelet use were higher in the present study. Of note, the bleeding risk of Group 3 in the present study expressed by the HAS-BLED score was elevated only modestly (2.3 ± 0.9) and comparable with that in the ELDERCARE-AF trial [7] and subanalysis of the ANAFIE Registry [9] (Supplementary Table 1).

Recently, several subanalyses using cluster analysis were reported from the Japanese AF registries [24–28]. The clusters in each registry most similar to Group 3 in our study were as follows: the atherosclerotic comorbid cluster in the J-RHYTHM Registry [25], the very elderly cluster in the Fushimi AF Registry [26], the high mortality- and heart failure-risk cluster in the Shinken Database [27], and the cluster of the very elderly patients with a high prevalence of previous major bleeding in the ANAFIE Registry [28]. The patients in these clusters had not only higher bleeding events but also higher all-cause mortality than those in the other clusters [25–28], as in Group 3 of the present study.

### Impact of high bleeding risk on adverse events in NVAF patients

As shown in Table 3 and Fig. 1, the rate of major hemorrhage in Group 3 was highest among the three groups, indicating that patients in Group 3 were certainly at a higher risk of bleeding events. Notably, the rate of ICH in Group 3 was comparable to that in Group 2 but higher than that in Group 1. In contrast, the rate of gastrointestinal bleeding in Group 3 was markedly higher than that in Group 2 (Table 3). These findings suggested that advanced age (≥ 80 years) itself was an important factor for the incidence of ICH, whereas other factors such as antiplatelet use, low body weight, and low CrCl could be more critical for the development of gastrointestinal bleeding.

In addition to major hemorrhage, the crude rates of thromboembolism, all-cause death, and cardiovascular death in Group 3 were the highest among the three groups. Accordingly, the unadjusted HRs for all adverse events in Group 3 were significantly higher than those in Group 1 (Table 4). However, after adjusting for confounding factors in the multivariable analysis, the significance of the HRs for thromboembolism, major hemorrhage, and cardiovascular death disappeared, and only the HR for all-cause death in Group 3 remained significantly higher than that in Group 1 (Table 4). These findings indicate that the components of high bleeding risk in the present analysis were strongly associated with all-cause death rather than bleeding events in elderly patients with NVAF. This seems reasonable, because low body weight and low CrCl were previously identified as risk factors for all-cause death [17, 18] and were not included in the conventional risk scores, such as the CHADS_2_, CHA_2_DS_2_-VASc, and HAS-BLED scores [15, 20, 21]. Accordingly, we propose that for the management of NVAF patients, physicians should be cautious of not only bleeding complications but also all-cause death comprehensively, when patients are very elderly and have high bleeding risks.

### High bleeding risk and anticoagulation therapy

As shown in Fig. 2, the incidence rate of major hemorrhage in patients treated with warfarin in Group 3 was apparently higher than that in those without anticoagulation therapy, although the difference was not statistically significant. Conversely, the rate of thromboembolism in patients treated with warfarin in Group 3 was numerically lower than that in those without anticoagulation therapy; however, the difference was also not statistically significant (Fig. 2). These findings indicate that anticoagulation therapy with warfarin might be effective in preventing thromboembolism even in very elderly patients with a high bleeding risk, whereas the risk of bleeding could be enhanced by warfarin. However, this was not robustly evident in this study because of the small number of patients in each subgroup of Group 3. Thus, we compared the incidence rates of thromboembolism and major hemorrhage with those of the ELDERCARE-AF trial [7] and the high-bleeding-risk group in subanalysis of the ANAFIE Registry [9] (Supplementary Figure). Although the overall event rates were lower in our study than in the ELDERCARE-AF trial, similar trends were observed in the incidence rates of thromboembolism and major hemorrhage in the high-bleeding-risk patients in the present study (Group 3) and in the other two studies (Supplementary Figure). In contrast, all-cause death in patients treated with warfarin in Group 3 was significantly lower than that in those without warfarin therapy (Fig. 3). These results suggested that warfarin might have beneficial effects on outcomes even in elderly NVAF patients with a high bleeding risk in our cohort of the warfarin era, as we previously reported in very elderly NAVF patients aged ≥ 85 years [29].

In a recent report from Asia using a largest observational cohort in Taiwan [30], warfarin use was significantly associated with a lower risk of ischemic stroke and positive net clinical benefit, without an increased risk of ICH, compared with no anticoagulation therapy, among very elderly AF patients aged ≥ 90 years (age, 96.2 ± 2.7 years; men, 39.2%; CHA_2_DS_2_-VASc score, 5.3 ± 1.6; history of major bleeding, 8.6%) in the warfarin era (1996–2011) [30]. All-cause mortality was not mentioned in that study [30]. In contrast, NOACs were significantly associated with a lower risk of ICH compared with warfarin in the NOAC era (2012–2015). According to the above study [30] and the ELDERCARE-AF trial [7], NOACs would be more favorable choice than warfarin as anticoagulation therapy in the very elderly NVAF patients with a high bleeding risk in the current NOAC era.

Further studies are warranted to validate event rates and the effect of oral anticoagulants on outcomes in very elderly NVAF patients with a high bleeding risk, as defined by the ELDERCARE-AF criteria, in the NOAC era.

## Limitation

This study has several limitations. First, it was a post hoc analysis of data from the J-RHYTHM Registry [11, 12] and was, therefore, hypothesis-generating in nature. Second, the study subjects were recruited from only 158 institutions in Japan, and most of the participating physicians specialized in cardiology and management of cardiac arrhythmias. Therefore, these results may not be generalizable to the entire Japanese population with NVAF. In addition, because all study participants were Japanese, these data may not necessarily be applicable to other racial/ethnic groups. Third, changes in drugs, dosages, and drug adherence during the follow-up period were not considered in the analysis. Fourth, NSAID use, frailty, dementia, or a history of fall was not evaluated, because these data were not collected; therefore, the high bleeding risk might have been underestimated. Fifth, although only patients with a CHADS_2_ score ≥ 2 were included in the ELDERCARE-AF trial based on its entry criteria [7], 67 patients aged ≥ 80 years with high bleeding risk and a CHADS_2_ score of 1 were included in Group 3 of the present study based on the current package insert of edoxaban, in which a CHADS_2_ score ≥ 2 is not an essential criterion for the use of once-daily 15-mg edoxaban in Japan. However, the influence of the 67 patients on adverse events might be small, because all event rates were comparable between the 597 patients in Group 3 and the 530 patients after excluding 67 patients with a CHADS_2_ score of 1 (Supplementary Table 2). Finally, data from the warfarin era were used for the present analysis. Therefore, caution should be exercised when extrapolating the present results to patients with NVAF in the NOAC era. In addition, although warfarin use was significantly associated with lower all-cause mortality in Group 3 (Fig. 3), causality cannot be determined from this study because of observational nature.

## Conclusions

The NVAF patients aged ≥ 80 years with one or more high bleeding risks were certainly at a higher risk of adverse events, especially all-cause death. A high bleeding risk as defined by the ELDERCARE-AF criteria was strongly associated with all-cause death rather than major hemorrhage in very elderly patients with NVAF.

### Supplementary Information

Below is the link to the electronic supplementary material.

Supplementary file1 (PDF 530 kb)

## Data Availability

The deidentified participant data will be shared on a request basis. Please directly contact the corresponding author to request data sharing.
