# Supplementary material for: Characteristics and outcomes in elderly patients with non-valvular atrial fibrillation and high bleeding risk: subanalysis of the J-RHYTHM Registry
Source: Heart Vessels. 2023 Dec 16;39(4):330–9. doi: 10.1007/s00380-023-02343-9 (PMC10920444; doi:10.1007/s00380-023-02343-9)
Supplement: Supplementary file 1 — Supplementary file1 (PDF 530 kb) [file 380_2023_2343_MOESM1_ESM.pdf]

Supplementary Table 1. Patient characteristics and medications in three studies

**Study (Group)**	**ELDERCARE -AF trial**[7)]	**ANAFIE Registry**[9)] **(High-risk group)**	**J-RHYTHM Registry (Group 3, ≥80 years with HBR)**
Number of patients	984	7104	597
Age, years	86.6±4.2	85.0±4.0	83.7±3.4
>85 years	537 (54.6%)	-	152 (25.5%)
Sex, men	419 (42.6%)	3019 (43.8%)	327 (54.8%)
Body weight, kg	50.6±11.0	51.2±11.1	51.4±11.0
**≤45 kg***	**374 (38.0%)**	**2687 (37.8%)**	**201 (33.7%)**
Body mass index, kg/m^2	22.1±3.7	-	21.3±3.6
Systolic BP, mmHg	-	126.2±18.1	125.8±17.3
Creatinine clearance, mL/min	36.3±14.4	35.4±13.9	37.1±13.8
**15–30 mL/min***	**403 (41.0%)**	**2856 (40.2%)**	**188 (31.5%)**
Type of atrial fibrillation			
Paroxysmal	463 (47.1%)	2844 (40.0%)	184 (30.8%)
Non-paroxysmal	521 (52.9%)	4260 (60.0%)	413 (69.2%)
Comorbidities			
Heart failure	533 (54.2%)	3689 (51.9%)	276 (46.2%)
Hypertension	810 (82.3%)	5841 (82.2%)	388 (65.0%)
Diabetes mellitus	225 (22.9%)	2037 (28.7%)	119 (19.9%)
Stroke/TIA	236 (24.0%)	2130 (30.0%)	132 (22.1%)
Coronary artery disease	257 (26.1%)	683 (9.6%)#	127 (21.3%)
Dementia	160 (16.3%)	948 (13.3%)	NE
Fall within 1 year	340 (34.6%)	760 (10.7%)	NE
$CHADS_2$ score	3.1±1.1	3.2±1.1	2.8±1.2
CHA_2DS_2-VASc score	4.9±1.3	-	4.4±1.3
HAS-BLED score	2.3±0.9	2.2±0.9	2.3±0.9
**History of bleeding***	**222 (22.6%)**	**740 (10.4%)**	**59 (9.9%)**
Anticoagulant	492 (50.0%)	6324 (89.0%)	470 (78.7%)
Warfarin	0 (0%)	2139 (30.1%)	470 (78.7%)
TTR†, %	-	71.0±31.3	66.3±25.5
NOAC	492 (50.0%)‡	4184 (58.9%)	0 (0%)
**Antiplatelet***	**529 (53.8%)**	**3024 (42.6%)**	**367 (61.5%)**
**NSAIDs***	**317 (323%)**	**-**	**NE**

Data are number of patients (%) or mean±standard deviation

HBR, high bleeding risk; BP, blood pressure; TIA, transient ischemic attack; $CHADS_2$, congestive heart failure, hypertension, age ≥75 years, diabetes mellitus, and history of stroke or TIA; CHA_2DS_2-VASc, additionally, vascular disease (coronary artery disease), age 65–74 years, and female sex; HAS-BLED, hypertension, abnormal renal/liver function, stroke, bleeding history or predisposition, labile INR (episodes of INR ≥3.5), elderly (age >65 years), drugs (use of antiplatelets)/alcohol concomitantly; INR, international normalized ratio; TTR, time in therapeutic range; NOAC, non-vitamin K antagonist oral anticoagulant; NSAIDs, anti-inflammatory drugs; NE, not evaluated.

*** Components of high bleeding risk**. # Only myocardial Infarction.

† Target INR was 2.0–3.0 (<70 years old) or 1.6–2.6 (≥70 years old). ‡ Only edoxaban 15 mg.

Supplementary Table 2. Crude event numbers and rates during two-year follow-up period in Group 3 and alternative Group 3

	**Group 3 (≥80 years with HBR including $CHADS_2$ score 1)**	**Alternative Group 3 (≥80 years with HBR and $CHADS_2$ score ≥2)**	***P*-Value**
Number of patients	597	530	
**Thromboembolism**	23 (3.9%)	18 (3.4%)	0.683
**Major hemorrhage**	22 (3.7%)	21 (4.0%)	0.808
Intracranial hemorrhage	9 (1.5%)	9 (1.7%)	0.799
Gastrointestinal bleeding	9 (1.5%)	8 (1.5%)	0.998
Other bleeding or unknown	4 (0.7%)	4 (0.8%)	0.866
**All-cause death**	51 (8.5%)	47 (8.9%)	0.847
**Cardiovascular death**	15 (2.5%)	15 (2.8%)	0.741

Data are number of patients (%).
HBR, high bleeding risk; $CHADS_2$, congestive heart failure, hypertension, age ≥75 years, diabetes mellitus, and history of stroke or transient ischemic attack.

Supplementary Figure

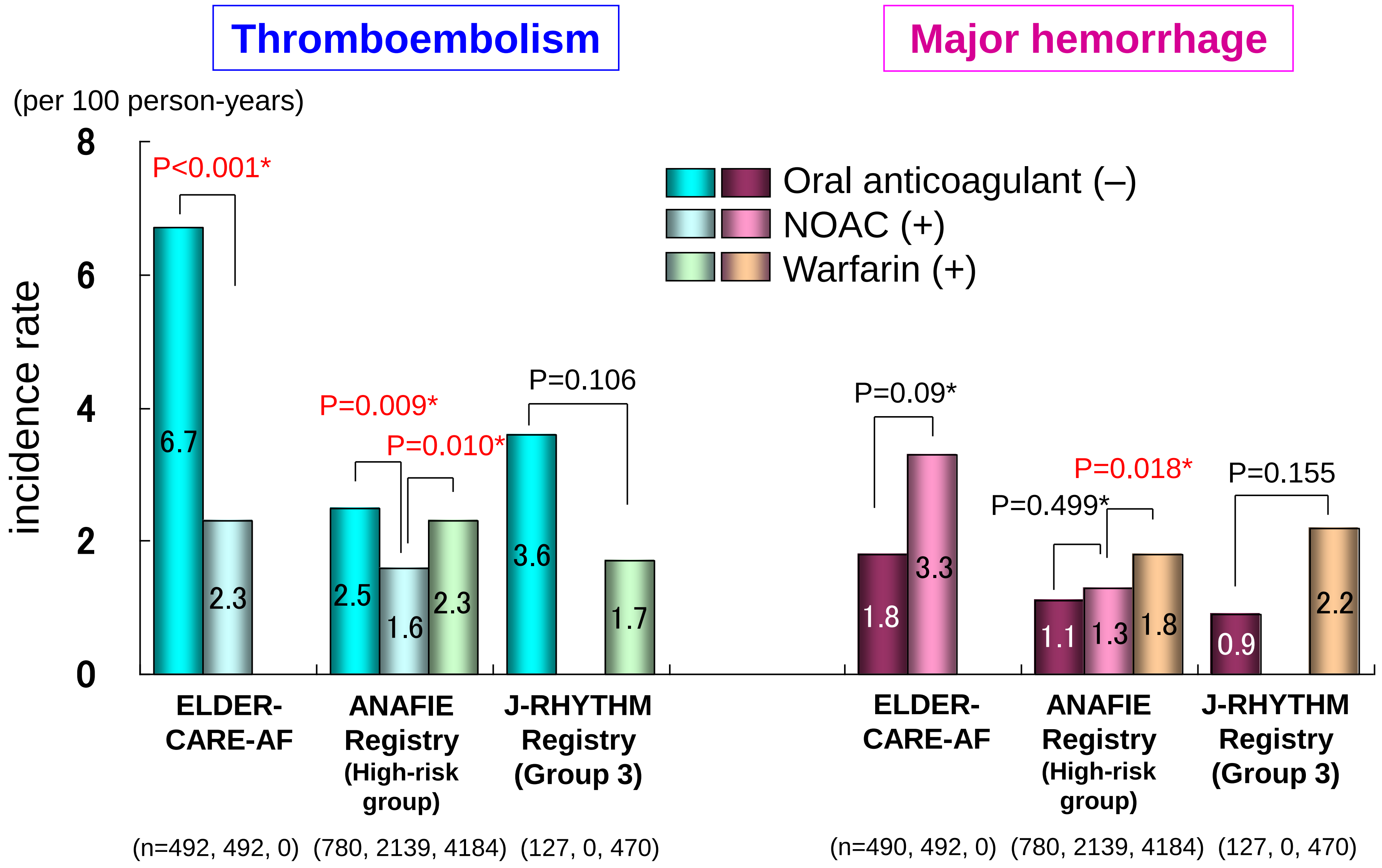

Supplementary Figure Legend

Supplementary Figure. Incidence rates of thromboembolism and major hemorrhage in the three studies.

P-values: comparison between two groups.

* P-values by the Cox proportional hazard model.

NOAC, non-vitamin K antagonist oral anticoagulant.

Generated from references [7] and [9].
